# High Content Analysis of Hippocampal Neuron-Astrocyte Co-cultures Shows a Positive Effect of Fortasyn Connect on Neuronal Survival and Postsynaptic Maturation

**DOI:** 10.3389/fnins.2017.00440

**Published:** 2017-08-04

**Authors:** Anne-Lieke F. van Deijk, Laus M. Broersen, J. Martin Verkuyl, August B. Smit, Mark H. G. Verheijen

**Affiliations:** ^1^Department of Molecular and Cellular Neurobiology, Center for Neurogenomics and Cognitive Research, VU University Amsterdam Amsterdam, Netherlands; ^2^Advanced Medical Nutrition, Nutricia Research Utrecht, Netherlands

**Keywords:** method, neuron-astrocyte co-culture, synapse formation, nutritional intervention, phospholipids

## Abstract

Neuronal and synaptic membranes are composed of a phospholipid bilayer. Supplementation with dietary precursors for phospholipid synthesis –docosahexaenoic acid (DHA), uridine and choline– has been shown to increase neurite outgrowth and synaptogenesis both *in vivo* and *in vitro*. A role for multi-nutrient intervention with specific precursors and cofactors has recently emerged in early Alzheimer's disease, which is characterized by decreased synapse numbers in the hippocampus. Moreover, the medical food Souvenaid, containing the specific nutrient combination Fortasyn Connect (FC), improves memory performance in early Alzheimer's disease patients, possibly via maintaining brain connectivity. This suggests an effect of FC on synapses, but the underlying cellular mechanism is not fully understood. Therefore, we investigated the effect of FC (consisting of DHA, eicosapentaenoic acid (EPA), uridine, choline, phospholipids, folic acid, vitamins B12, B6, C and E, and selenium), on synaptogenesis by supplementing it to primary neuron-astrocyte co-cultures, a cellular model that mimics metabolic dependencies in the brain. We measured neuronal developmental processes using high content screening in an automated manner, including neuronal survival, neurite morphology, as well as the formation and maturation of synapses. Here, we show that FC supplementation resulted in increased numbers of neurons without affecting astrocyte number. Furthermore, FC increased postsynaptic PSD95 levels in both immature and mature synapses. These findings suggest that supplementation with FC to neuron-astrocyte co-cultures increased both neuronal survival and the maturation of postsynaptic terminals, which might aid the functional interpretation of FC-based intervention strategies in neurological diseases characterized by neuronal loss and impaired synaptic functioning.

## Introduction

During neuronal development, neurites sprout from the cell body and elongate, followed by the formation of dendrites and axons and the formation of synapses, allowing synaptic transmission and plasticity (Govek et al., 2005; Wurtman et al., 2009b). Synapses contribute in important ways to learning and memory processes. In neurodegenerative diseases, such as Alzheimer's disease (AD), decreased synapse density correlates with memory and cognitive impairments (Terry et al., 1991; Masliah et al., 2001; Selkoe, 2002). Neuronal and synaptic membranes consist of a bilayer of lipids, like all cellular membranes, but with characteristic high levels of cholesterol and phospholipids, especially phosphatidylcholine (PC), and polyunsaturated fatty acids (PUFAs) (Takamori et al., 2006; Puchkov and Haucke, 2013). Phospholipids are synthesized by the Kennedy pathway which requires the precursors docosahexaenoic acid (DHA; an omega-3 PUFA), uridine and choline (Kennedy and Weiss, 1956; Wurtman et al., 2009a). Several studies have shown that supplementation with these phospholipid precursors enhances neurite outgrowth and synaptogenesis both *in vivo* and *in vitro*. For instance, it was shown that oral administration of a combination of uridine-5′-monophosphate (UMP; a source of uridine) and DHA in rats and gerbils increased hippocampal spine density and synaptic protein levels (Sakamoto et al., 2007; Cansev et al., 2009). Furthermore, dietary supplementation with a combination of uridine, DHA and choline improved spatial learning and memory in healthy gerbils (Holguin et al., 2008). Previously, it has been demonstrated that DHA supplementation enhanced neurite outgrowth in cultured rat hippocampal neurons (Calderon and Kim, 2004).

Interestingly, phospholipid precursors and cofactors have successfully been used for intervention in patients with early AD. These patients had improved memory performance after consuming Souvenaid, a medical food that contains Fortasyn Connect (FC), which comprises DHA, eicosapentaenoic acid (EPA), uridine, choline, phospholipids, folic acid, vitamins B12, B6, C and E, and selenium) (Scheltens et al., 2010, 2012; De Waal et al., 2014). It was suggested that FC increased functional neuronal connectivity in these AD patients (De Waal et al., 2014), stressing the importance of understanding how FC affects synapse formation and maturation. Astrocytes are important contributors to neuronal lipid supply, and contribute to the formation, maturation and maintenance of synapses, thereby metabolically supporting neuronal communication (Mauch et al., 2001; Pfrieger, 2003). The aim of the current study was to determine the effect of FC on synaptogenesis, by using co-cultures of primary isolated hippocampal neurons and astrocytes, which were screened and analyzed using the Opera High Content Screening system. Using a newly developed data analysis, we measured neuronal developmental processes, i.e., neuronal survival and neurite morphology, as well as the formation and maturation of synapses. The combined supplementation with nutritional phospholipid precursors and cofactors, as provided by FC, to neuron-astrocyte co-cultures, resulted in increased neuronal survival and increased synaptic maturation of postsynaptic terminals.

## Materials and methods

### Neuron-astrocyte co-culture

All experimental procedures involving animals were approved by the local animal research committee (Dierexperimentencommissie VU University) and complied with the European Council Directive (86/609/EEC). Cortical astrocytes were collected from P1 wild-type mice (C57/BL6 breeders were obtained from Charles River and were bred in the animal facility of the VU University Amsterdam). Cortices were dissected, cleared of meninges and collected in ice-cold Hanks Buffered Salt Solution (HBSS; Sigma-Aldrich) buffered with 7 mM HEPES (pH 7.4; Invitrogen). The tissue was mechanically fragmented and incubated in HBSS, HEPES and 0.25% trypsin (Invitrogen) at 37°C for 30 min. Trypsinization was quenched by adding astrocyte culture medium: Dulbecco's modified Eagle's medium + GlutaMAX (Gibco) supplemented with MEM non-essential amino acids solution (Sigma), 1% penicillin-streptomycin (Invitrogen) and 10% fetal bovine serum (Gibco). Subsequently, the tissue was centrifuged at 1200 rpm for 10 min, the pellet resuspended and cells were plated in astrocyte culture medium in poly-L-lysine-coated (Gibco) T75 flasks and kept in a 37°C/5% CO_2_ incubator. After reaching confluence, cells were dissociated with 1.25% trypsin in phosphate buffered saline (PBS, pH 7.4; Invitrogen) for 4 min. Trypsinization was quenched by adding astrocyte culture medium. Cells were spinned down at 800 rpm for 5 min, the pellet was resuspended and cells were counted. Astrocytes were seeded on a poly-D-lysine/laminin-coated 96-well plate at a density of 10K/well. When confluence was reached, the astrocyte culture medium was removed, cells were washed two times with PBS and NBM+B-27 was added: Neurobasal medium (Gibco) supplemented with 1x B-27 (Gibco), 1.8% HEPES (Invitrogen), 5 mM glutamax (Invitrogen) and 0.1% penicillin-streptomycin. Two days later, primary hippocampal neurons were added to the astrocyte monolayer. In brief, hippocampi were collected from E18 wild-type Wistar rats (Charles River). Hippocampi were incubated in HBSS with HEPES and 0.25% trypsin at 37°C for 30 min. Tissue was washed twice with HBSS and HEPES to remove trypsin. Subsequently, cells were dissociated by repeated trituration through a fire-polished Pasteur pipet. Hippocampal neurons (5K/well) were plated in 50% fresh NBM+B-27 and 50% 2-day astrocyte conditioned NBM+B-27 medium. Cells were cultured and supplemented according to a supplementation protocol (see “nutritional supplementation”).

### Nutritional supplementation

The following concentrations of FC components were present in the FC stock solution (1,000x stock; (Savelkoul et al., 2012)): docosahexaenoic acid (DHA; 14.4 mM), eicosapentaenoic acid (EPA; 10.1 mM), uridine (50 mM), choline chloride (20 mM), vitamin B6 (pyroxidine; 10 mM), vitamin B12 (100 μM), vitamin B9 (folic acid; 15 mM), phosphatidylcholine (PC; 25 mM), vitamin C (ascorbic acid; 75 mM), vitamin E (alpha-tocopherol; 20 mM), and selenium (sodium selenite; 400 μM). Stock components DHA and EPA were dissolved in absolute ethanol and diluted 5 times in fatty acid free bovine serum albumin (FAF-BSA; final concentration of 0.375 mM), PC and vitamin E were dissolved in absolute ethanol, folic acid in 1 M NaOH and vitamin B6 in 1 M HCl, prior to supplementation. All other stock components were dissolved in demineralized water. The concentration series of the total FC stock combination and of solvent mixtures were tested to check for cell viability (data not shown). Experiments were continued with dilution of the 1x FC stock (Savelkoul et al., 2012): 0.05x (1:20 of the 1x stock), 0.1x (1:10) and 0.2x (1:5). Results of FC supplementation were compared to their corresponding solvent condition (containing the same concentration of ethanol, FAF-BSA, NaOH and HCl present in the FC nutrient mix). Per condition, 8–12 wells were imaged.

Hippocampal neurons co-cultured with astrocytes were plated as described above. After 5 days in NBM-B-27, the medium was removed and cultures were gently washed twice with 0.1 M PBS to remove remaining medium. Neurobasal medium supplemented with 1x N2 (Gibco), 1.8% HEPES, 5 mM glutamax and 0.1% penicillin-streptomycin, referred to as NBM-N2, was then added, with or without FC or solvents. At day 12, half of the medium was replaced with fresh NBM-N2, with or without FC or solvents. After 14 days, cells were prepared for immunohistochemistry.

### Immunohistochemistry

After fixation with 4% paraformaldehyde (PFA) and 4% sucrose in 0.1 M PBS for 10 min, cells were washed two times with 0.1 M PBS followed by blocking in blocking solution (3% bovine serum albumin and 0.2% Triton X-100 in 0.1 M PBS) for 30 min. Cells were incubated with primary antibodies in blocking solution overnight at 4°C, subsequently washed four times with 0.1 M PBS and incubated in blocking solution with the appropriate secondary antibodies for 1 h at room temperature. Cells were washed then three times with 0.1 M PBS and one time with water before incubation with Hoechst (1:20,000, Molecular Probes) to counterstain DNA for 15 min. Lastly, wells were washed two times with water and were ready for analysis. Primary antibodies were rabbit anti-synapsin1 (1:1,000, Millipore), mouse anti-PSD95 (1:250, Thermo Scientific) and chicken anti-MAP2 (1:5,000, Bio-connect). Secondary antibodies were goat anti-rabbit Alexa 568 (1:400, Molecular Probes), goat anti-mouse Alexa 488 (1:400, Molecular Probes) and goat anti-chicken Alexa 647 (1:400, Molecular Probes).

### High-content screening

Confocal images were taken with the Opera High Content Screening system (PerkinElmer) and analyzed with the Columbus software (version 2.5.2.124862, PerkinElmer).

#### Neurite morphology and synaptic spots

To analyze cell survival, neurite morphology and synaptic spots, images were taken with a 20x objective (40 images per well) at a fixed focal plane. The following parameters were assessed: number of astrocyte and neuronal nuclei, neurite length, number of neurites and neurite branch points, and number of synapses per well and per neurite length (total number of synapses divided by the total neurite length). The analysis was done as follows: (1) Hoechst-positive nuclei were detected, including both neuronal and astrocyte nuclei. A training algorithm was designed to determine a cutoff that was used to distinguish neuronal nuclei from astrocyte nuclei. During the training phase, the researcher manually categorized cells into either neurons or astrocytes (at least 20 cells per category). In general, neuronal nuclei are characterized by higher nuclear MAP2 intensity and nuclei are smaller with higher Hoechst intensity compared to astrocyte nuclei. Properties of the selected cells (e.g., nucleus size, nucleus staining intensity and MAP2 staining intensity within the nucleus) were determined and using a linear classifier, the best linear combination of input properties was found yielding the best separation of the two classes.

Nuclei which were not MAP2-positive and which were too small to belong to astrocytes were defined as “remaining cells.” (2) MAP2-positive neurites were detected using neuronal nuclei as starting point. In this way, erroneous tracing of neuronal neurites in astrocytes, due to MAP2 background staining in astrocytes, was eliminated. Furthermore, this made it possible to decrease the threshold for neurite detection, leading to a better neurite tracing, even of thin and less intense neurites. Thus, only fields containing neuronal nuclei were included in further analysis. (3) The neurite tree was resized by shifting the neurite border with 1.615 μm (2.5 pixels) in order to detect synapsin1-positive presynaptic spots laterally located along neurites. (4) Within the same resized neurites, PSD95-positive postsynaptic spots were detected. (5) Mature synapses were detected and counted. Mature spots were defined as presynaptic spots when appearing in close proximity of postsynaptic spots [within 0.646 μm (1 pixel)]. (6) Lastly, the remaining non-opposing pre- and postsynaptic spots were detected and defined as immature pre- and post-synaptic spots.

#### Synapse morphology

To analyze spot morphology, 100 images per well were taken with a 40x objective at a fixed focal plane. Pre- and post-synaptic puncta with a relative spot intensity of 0.145 or higher were considered as bona fide synaptic spots. The following parameters considering the morphology of presynaptic and postsynaptic spots, both immature and mature, were analyzed using steps 1–6 as described above: spot size (the total number of pixels within spot border), spot total intensity (the intensity of all pixels within the spot border) and spot mean intensity (the average intensity of a pixel within a spot: the total intensity divided by the number of pixels within the spot border).

For frequency distribution graphs, all individual spot intensity data were collected. The spot intensity parameter outcomes were divided over four classes; each frequency was determined and expressed as a percentage of the corresponding spot number.

### Statistical analysis

#### Outlier removal

Outlier removal was performed when the average of a well deviated by more than 2 standard deviations from the mean. For images taken with a 20x objective: data “per well” (neuronal survival) and “per neuron” (neurite outgrowth), were treated separately. When one well showed an outlier in a group, data of all parameters of that well in the same group were deleted. Concerning the spot morphology parameters: once an outlier was detected in one of the spot morphology parameters (spot size, total or mean intensity) of a spot category (immature or mature pre- or postsynaptic spot), the values of all spot morphology parameters of that particular spot category were deleted.

#### Statistical analysis

Since FC components were dissolved in solvents that could have a positive effect on neuronal differentiation and synaptogenesis, the results of supplementation with FC were always compared to the results of solvent supplementation. Therefore, statistical comparisons were performed using a two-way ANOVA followed by a *post-hoc* Student's *t*-test for each FC dilution and corresponding solvent condition, or for comparison of different FC or solvent dilutions.

## Results

**Figure 1:**
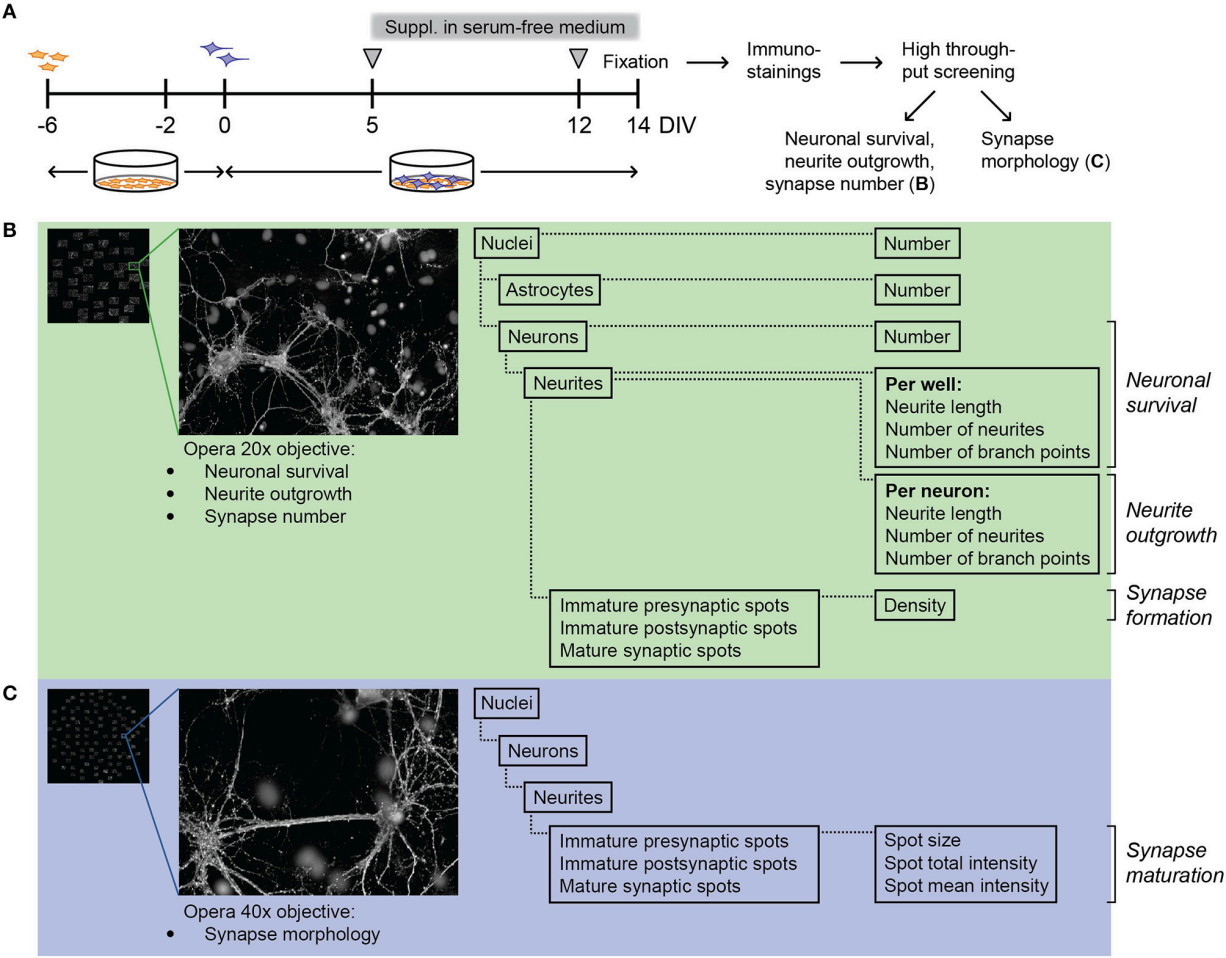
Experimental design of neuron-astrocyte co-cultures and high content screening. **(A)** Time line of culturing and supplementation of FC or solvents to neuron-astrocyte co-culture. Freshly isolated P1 mouse astrocytes (in orange) were plated and cultured in DMEM. After 4 days (DIV-2), DMEM was replaced by neurobasal medium with B27 (NBM+B27). Two days later (DIV0), half of the NBM+B27 was replaced by fresh NBM+B27 containing E18 rat hippocampal neurons (purple). After 5 days *in vitro* (DIV5), NBM+B27 was removed, cells were washed and serum free medium (NBM+N2) with or without FC or solvents was added. At DIV12, half of the medium was replaced by fresh serum-free medium with or without FC or solvents. Cells were fixed at DIV14, stained for nuclei, neurites, pre- and post-synaptic spots followed by Opera High Content Screening (HCS) to analyze cell survival, neurite morphology and synapse number (explained in more detail in **B**) as well as synapse morphology (explained in more detail in **C**). **(B)** Left: overview image showing 40 fields of one well taken by HCS with 20x objective. Close-up: example image of one imaged field. Columbus software was used to analyze cell number, neurite morphology and number of immature presynaptic spots, immature postsynaptic spots and mature synaptic spots. **(C)** Left: overview image showing 100 fields of one well taken by HCS with 40x objective. Close-up: example image of one imaged field. Columbus software was used to analyze spot size and intensity as a measure of synapse maturation.

To evaluate the effect of FC on synaptogenesis, FC was supplemented to primary neuron-astrocyte co-cultures after 5 days *in vitro* (DIV5; Figure 1A). At that time, most neurites are formed and synaptogenesis starts to occur, which makes it a suitable time point to study synapse formation and maturation (Pennypacker et al., 1991; Kim and Lee, 2012; Harrill et al., 2015). At DIV 14, co-cultures were imaged with the confocal Opera High Content Screening system to analyze neuronal survival, neurite morphology, and synaptic spots (Figure 1B), and synapse morphology (Figure 1C).

### Cell survival and neurite morphology

**Figure 2:**
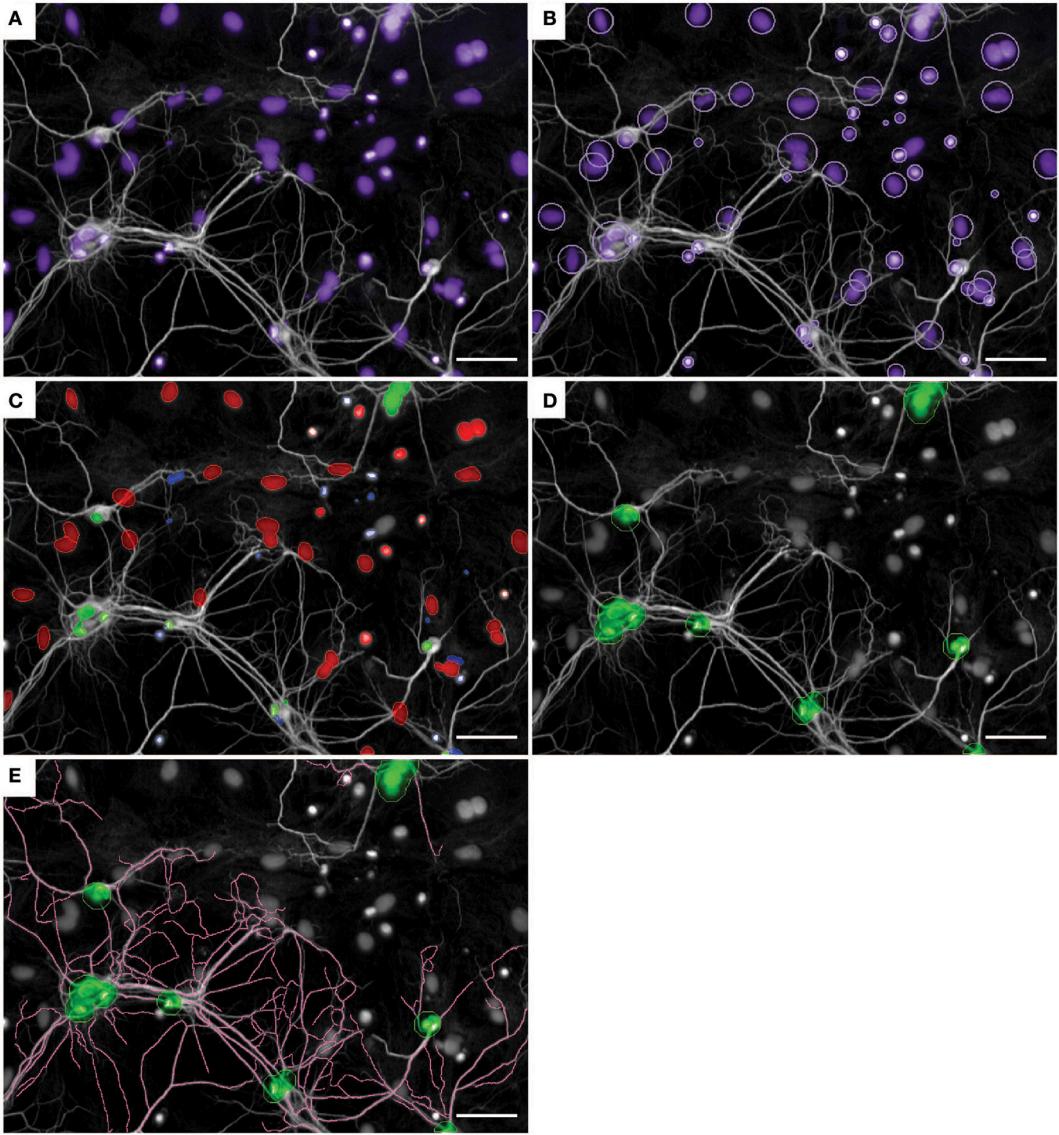
Nuclei and neurite detection in primary neuron-astrocyte co-cultures using Columbus software. **(A)** Input example image of co-culture in serum-free medium without supplementation. In white: MAP2-postive neurites, in purple: Hoechst-positive nuclei. **(B)** Nuclei detection based on Hoechst intensity. **(C)** Nuclei were categorized into three cell types: neurons (green), astrocytes (red), remaining cells (blue). **(D)** Neuronal nuclei are resized to improve neurite detection. **(E)** Neurite detection starting from resized neuronal nuclei. Images were taken with a 20x objective. Scale bar represents 50 μm.

To determine cell survival and neurite morphology, we analyzed the number of astrocytes and neurons with MAP2-positive neurites extending from the soma (Figure 2). Nuclei were categorized into either astrocyte or neuron nuclei based on the size and intensity of the Hoechst-positive nuclei as well as the intensity of the MAP2 signal surrounding the nuclei (Figure 2C). Next, neuronal nuclei were resized (Figure 2D) to enhance the tracing accuracy of MAP2-positive neurites that start at the nucleus (Figure 2E).

#### Neuronal survival

**Figure 3:**
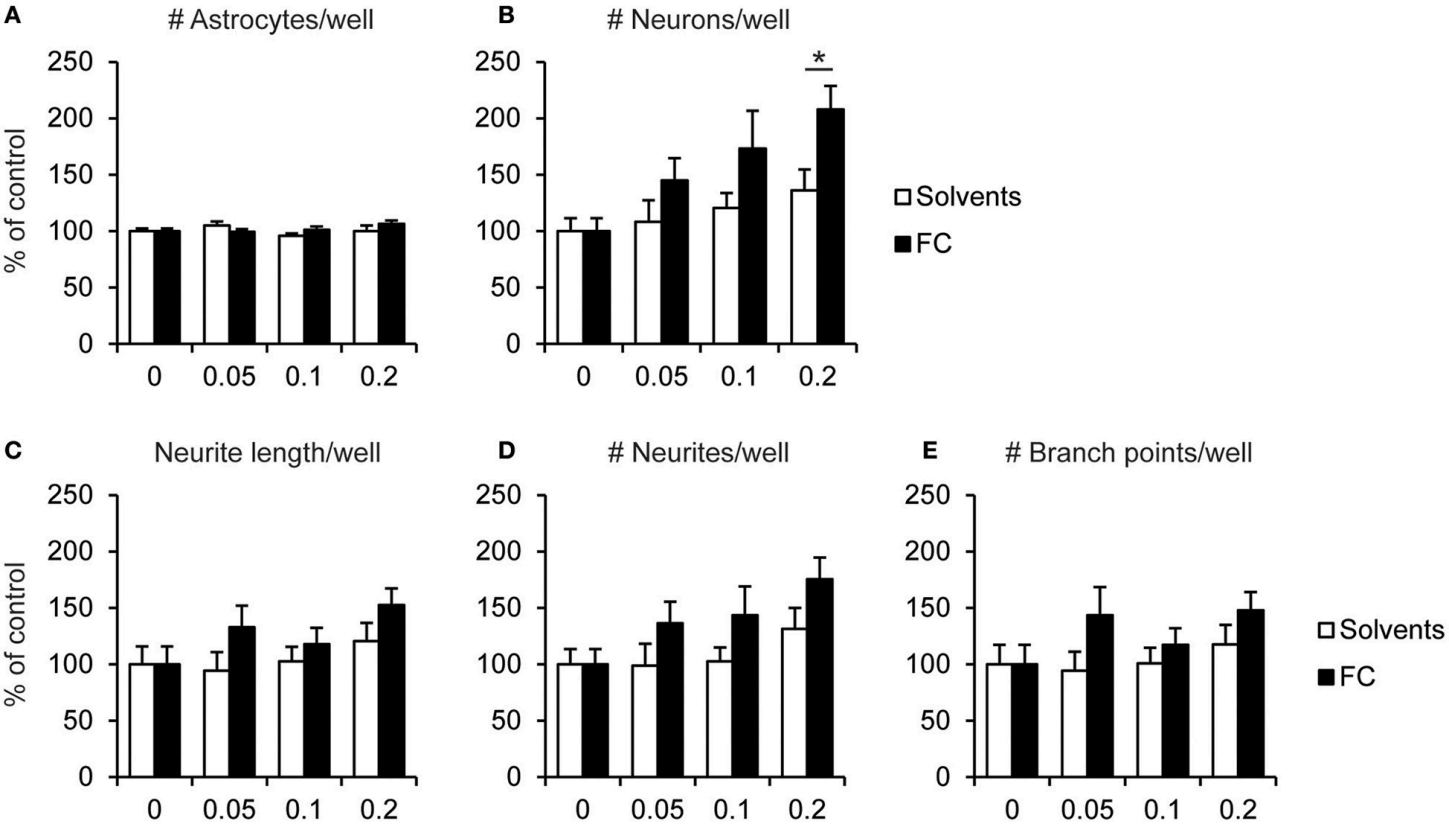
Effects of FC on cell survival and neurite morphology in primary neuron-astrocyte co-cultures. Analysis of **(A)** number of astrocytes, **(B)** number of neurons, **(C)** total neurite length, **(D)** number of neurites, **(E)** number of branch points measured per well for: no supplementation (0) or supplementation of 0.05x, 0.1x, or 0.2x FC or solvents (*n* = 7–12 wells per condition). Data were normalized to non-supplemented condition (0). Error bars represent standard error of the mean (SEM). ^*^p < 0.05.

Supplementation with 0.2x FC increased the number of neurons by 65% compared to the corresponding solvents supplementation [Figure 3B; *t*_(13)_ = 2.60, *p* = 0.022], whereas no effect of FC was observed on the number of astrocytes (Figure 3A). No significant effects were found for 0.2x FC on total neurite length [Figure 3C; *t*_(13)_ = 1.45, *p* = 0.172], the total number of branch points [Figure 3E; *t*_(13)_ = 1.26, *p* = 0.229] or the total number of neurites per well [Figure 3D; *t*_(13)_ = 1.65, *p* = 0.122]. These observations indicate that FC increased neuronal survival. The effect of FC on the lower number of neurites per neuron may be explained by its effect on increased neuronal cell number and thereby the increased formation of neuronal connectivity, which might decrease the need for the formation of extra neurites.

#### Neurite morphology

**Figure 4:**
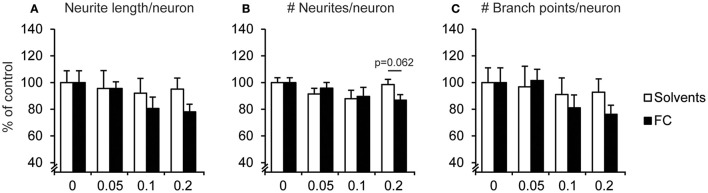
Effect of FC on neurite morphology in primary neuron-astrocyte co-cultures. Analysis of **(A)** neurite length, **(B)** number of neurites, **(C)** number of branch points per neuron for: no supplementation (0) or supplementation of 0.05x, 0.1x, or 0.2x FC or solvents (*n* = 7–11 wells per condition). Data were normalized to non-supplemented condition (0). Error bars represent standard error of the mean (SEM).

Neurite morphology was analyzed by measuring the number of neurite extensions from the soma, neurite length, and branches per neuron. No significant changes were found in neurite morphology after FC supplementation (Figure 4), which was as expected since supplementation started after most neurites had been formed (DIV5). For 0.2x FC supplementation, that strongly increased neuronal cell number, a trend toward decreased number of neurites per neuron was found [Figure 4B; *t*_(13)_ = −2.04, *p* = 0.062), although not for the neurite length per neuron [Figure 4A; *t*_(13)_ = −1.58, *p* = 0.138], nor for the number of branch points per neuron [Figure 4C; *t*_(13)_ = −1.34, *p* = 0.203].

### Synapse detection and quantification

**Figure 5:**
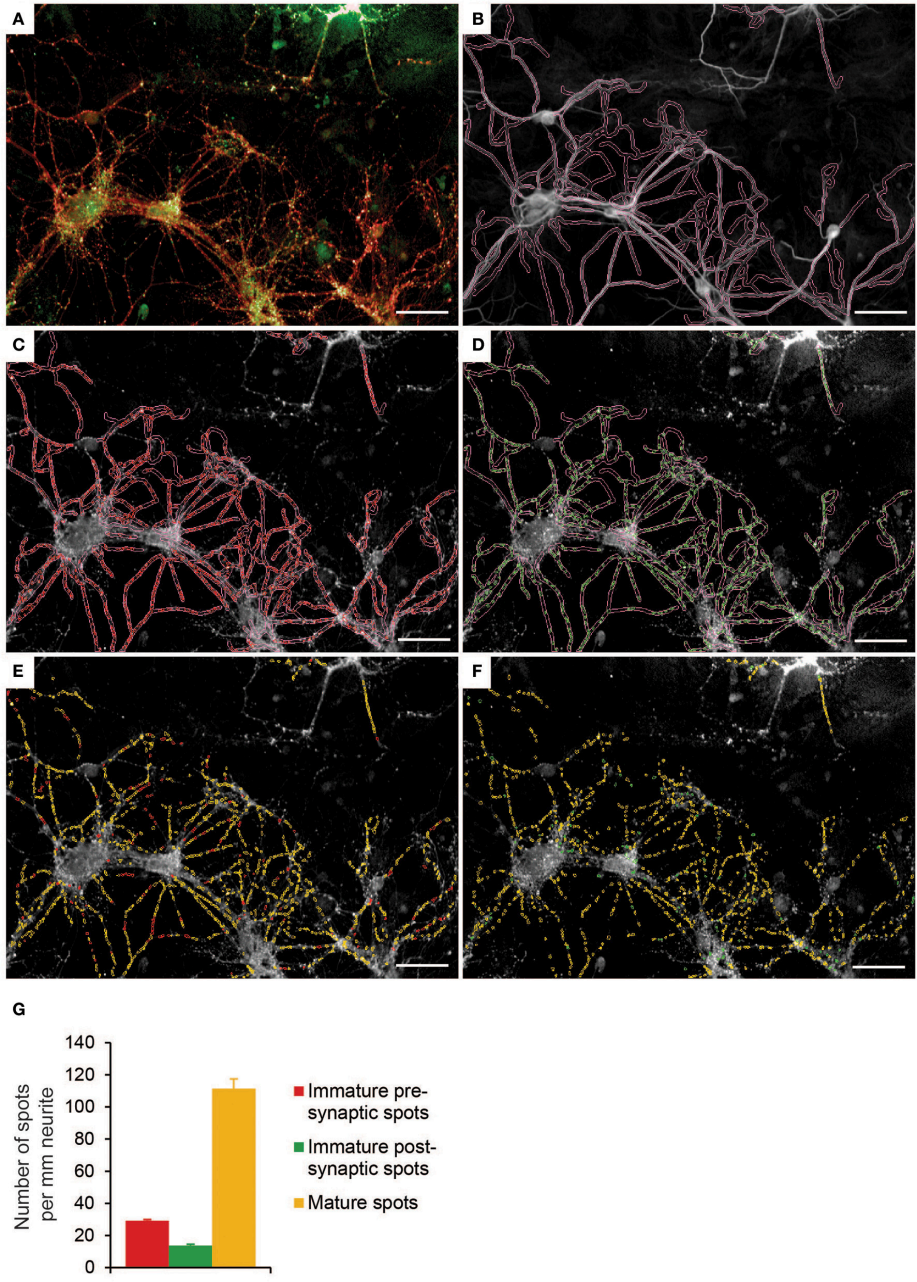
Synapse detection in a primary neuron-astrocyte co-culture using Columbus software. **(A)** Example image of control condition showing presynaptic spots (red) and postsynaptic spots (green). **(B)** Resized neurites. **(C)** Detection of presynaptic spots. **(D)** Detection of postsynaptic spots. **(E)** Presynaptic spots are categorized into either immature spots (red) or mature spots (yellow). Spot is mature when pre- and post-synaptic spots are in close proximity (2px). **(F)** Postsynaptic spots are categorized into either immature spots (green) or mature spots (yellow). **(G)** Quantification of number of mature spots, immature pre- and post-synaptic spots per mm neurite in control condition (*n* = 12 wells per condition). Error bars represent standard error of the mean (SEM). Images are taken with a 20x objective. Scale bar represents 50 μm.

Synaptogenesis involves the pre-patterning of both pre- and post-synaptic terminals, contact stabilization and synapse maturation (Shen and Cowan, 2010). Here we detected synapsin1-positive presynaptic spots and PDS95-positive postsynaptic spots along traced MAP2-positive neurites (Figures 5A–D). Opposed synapsin1/PSD95 spots were considered mature synapses, whereas not opposed synapsin1 or PSD95 spots were defined as immature presynaptic or immature postsynaptic spots, respectively (Figures 5E,F). We found that under basal culture conditions, most of the detected spots were mature spots (71%) and only a small fraction were immature presynaptic (20%) or immature postsynaptic (9%) spots (Figure 5G).

**Figure 6:**
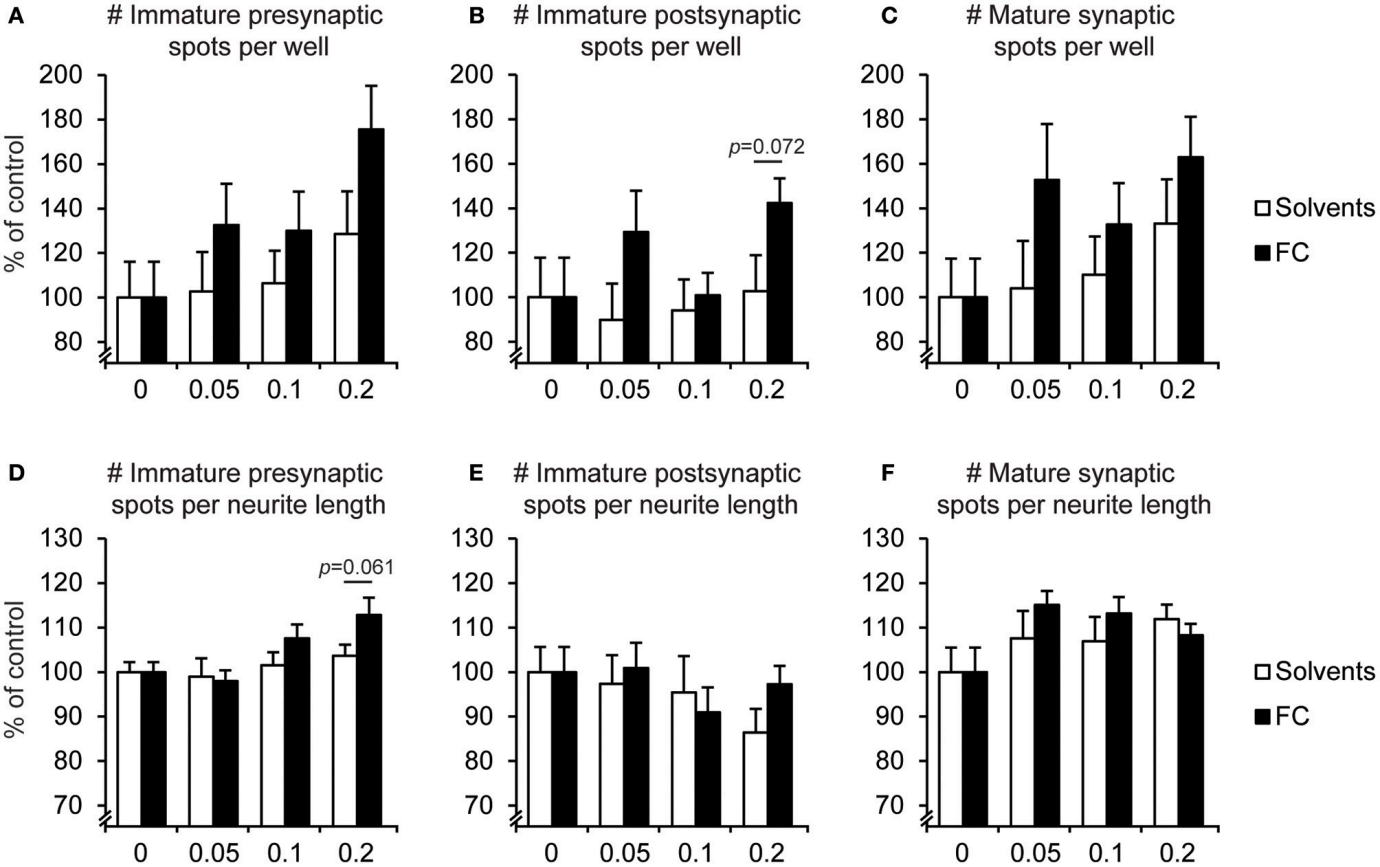
Effect of FC on synaptic spots in primary neuron-astrocyte co-cultures. Analysis of number of **(A,D)** immature presynaptic spots, **(B,E)** immature postsynaptic spots, **(C,F)** mature synaptic spots, per well **(A–C)** and per neurite length **(D–F)** for: no supplementation (0) or supplementation of 0.05x, 0.1x, or 0.2x FC or solvents (*n* = 6-11 wells per condition). Data were normalized to non-supplemented condition (0). Error bars represent standard error of the mean (SEM).

The effects of FC supplementation on synaptic spots are depicted in Figure 6. Supplementation with 0.2x FC slightly increased the relative amount of immature presynaptic spots [Figure 6D; *t*_(13)_ = 2.05, *p* = 0.061], without changing the small category of immature postsynaptic spots (Figure 6E). Furthermore, no effects were found for FC on the relative amount of mature spots.

### Synapse maturation

**Figure 7:**
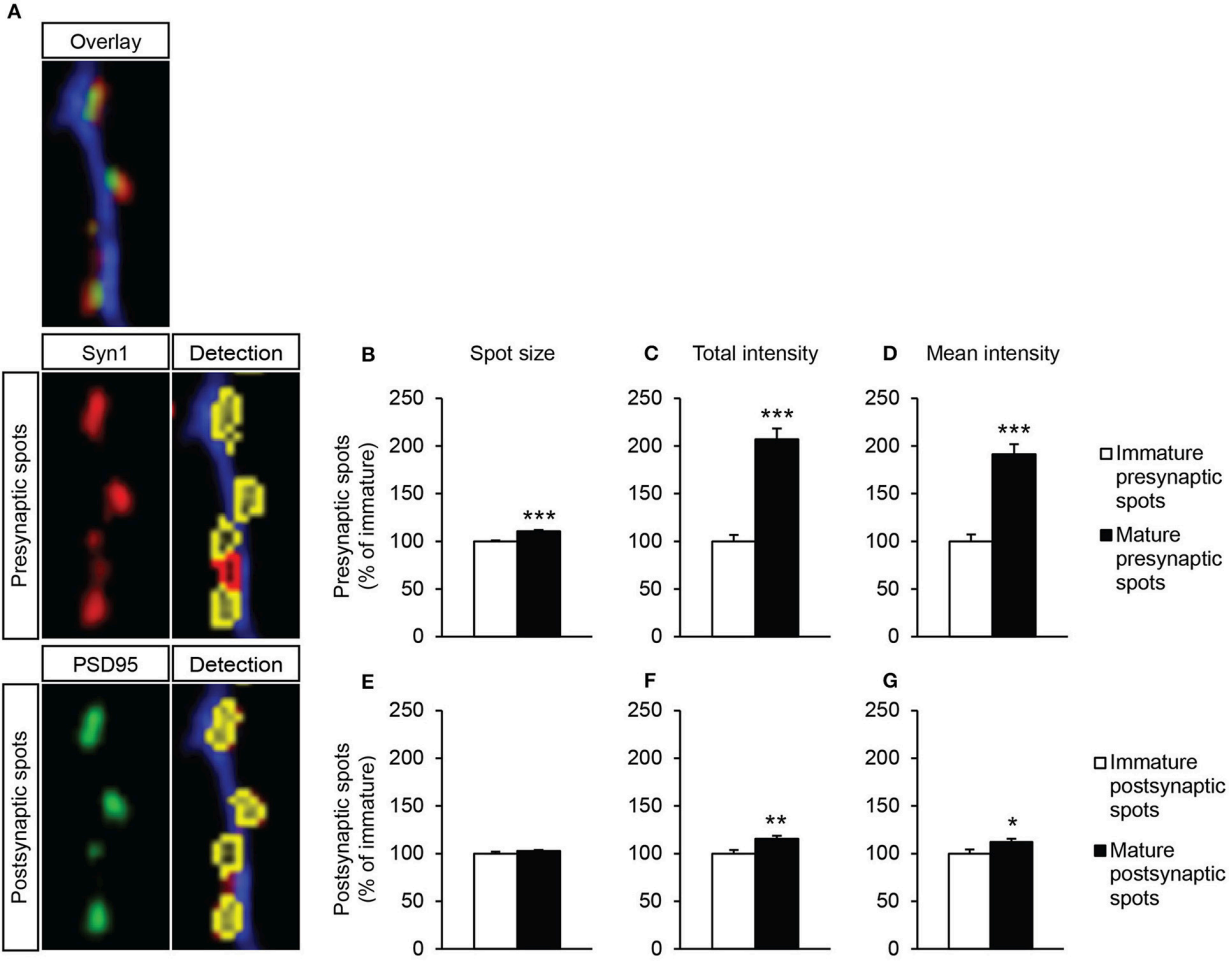
Synapse morphology analysis of non-supplemented neuron-astrocyte co-cultures by Columbus software. **(A)** Zoomed-in example images taken with 40x objective. In red: synapsin1-positive presynapses, in green: PSD95-positive postsynapses. Both are followed by detection of immature presynapses (red) or mature synapses (yellow). **(B–D)** Analysis of presynaptic spots. **(E–G)** Analysis of postsynaptic spots. **(B,E)** Spot size. **(C,F)** Total intensity of all pixels within a spot. **(D,G)** Intensity corrected for spot size (per spot: total intensity/μm^2^ spot area). Data were normalized to immature spot condition. Error bars represent standard error of the mean (SEM), *n* = 11 wells per condition, ^*^p < 0.05, ^**^p < 0.01, ^***^p < 0.001.

To gain more insight into synapse maturation, images taken at a higher magnification were analyzed for parameters of synapse morphology, e.g., spot area, total spot intensity and the spot intensity corrected for spot size (mean spot intensity). Since a large number of *in vivo* studies have described morphology differences between mature and immature synapses (Dyson and Jones, 1980; Ziv and Garner, 2004; Petralia et al., 2005), we first aimed to determine possible differences in spot morphology of immature and mature spots, under our non-supplemented *in vitro* condition (Figure 7A). Indeed, mature presynaptic spots were larger and twice as intense as immature presynaptic spots [Figure 7B; *t*_(19)_ = −6.03, *p* = 0.000, Figure 7C; *t*_(19)_ = −8.12, *p* = 0.000]. Moreover, the intensity corrected for the spot size was also strongly increased in mature presynaptic spots compared to immature presynaptic spots [Figure 7D; *t*_(19)_ = −7.05, *p* = 0.000]. No changes in spot size between immature and mature postsynaptic spots were found [Figure 7E; *t*_(21)_ = −1.21, *ns*]. However, mature postsynaptic spots were more intense also when corrected for spot size [Figure 7F; *t*_(21)_ = −3.14, *p* = 0.005, Figure 7G; *t*_(21)_ = −2.30, *p* = 0.032]. This demonstrates that our *in vitro* analysis method is able to determine differences between mature and immature synapse morphology, as has been described *in vivo*.

**Figure 8:**
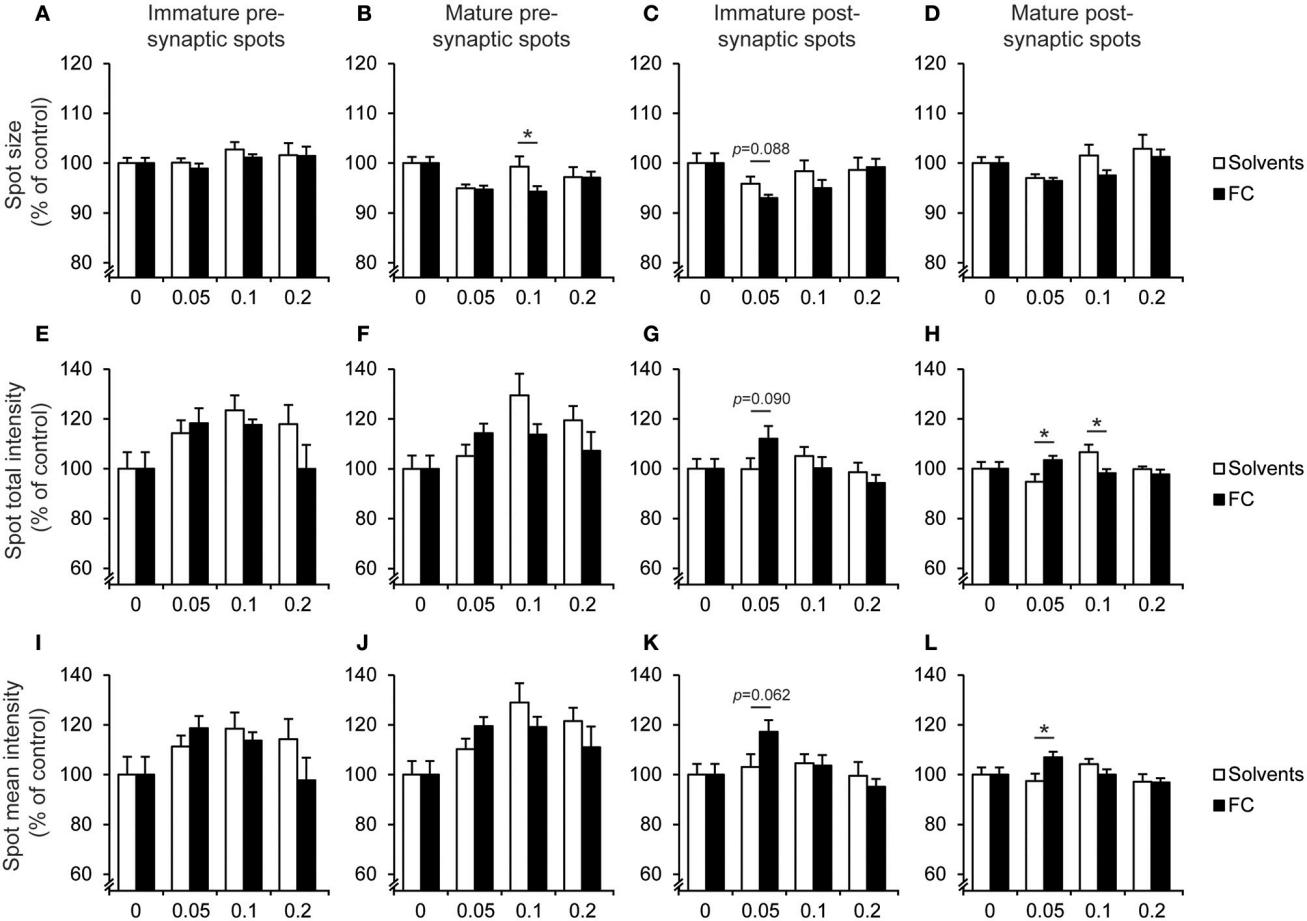
Effect of FC and solvent supplementation on spot morphology of hippocampal neurons co-cultured with astrocytes. Analysis of the effect of supplementation of 0.05x, 0.1x, 0.2x FC or solvents on the morphology of presynaptic immature **(A,E,I)** and mature **(B,F,J)** spots as well as postsynaptic immature **(C,G,K)** and mature **(D,H,L)** spots. Morphology was determined according to the parameters spot size **(A–D)**, total intensity of the spot **(E–H)** and intensity corrected for spot size **(I–L)**. Data were normalized to non-supplemented condition (0). Error bars represent standard error of the mean (SEM), *n* = 6–11 wells per condition, ^*^p < 0.05.

Next, the effect of FC on synapse morphology was determined (Figure 8). When compared to solvent supplementation, 0.05x FC supplementation significantly increased the total intensity of mature postsynaptic spots [Figure 8H; *t*_(14)_ = 2.44, *p* = 0.029], as well as the mean intensity [Figure 8L; *t*_(14)_ = 2.62, *p* = 0.02]. Additionally, trends were found for the effects of 0.05x FC on decreasing the size [Figure 8C; *t*_(14)_ = −1.83, *p* = 0.088], increasing the total intensity [Figure 8G; *t*_(14)_ = 1.82, *p* = 0.09] and increasing the mean intensity [Figure 8K; *t*_(14)_ = 2.03, *p* = 0.062] of immature postsynaptic spots. Thus, FC increased PSD95 levels in both mature and immature postsynaptic terminals, whereas presynaptic spot morphology remained unaffected. However, we observed that solvents also affected synapse morphology. Supplementation with 0.1x solvents increased the size of mature presynaptic spots [Figure 8B; *t*_(13)_ = −2.23, *p* = 0.044] when compared to 0.1x FC. Additionally, 0.1x solvent supplementation increased total intensity of mature postsynaptic spots [Figure 8H; *t*_(13)_ = −2.48, *p* = 0.028] when compared to supplementation with 0.1x FC. This suggests that 0.1x solvent supplementation leads to changes in the size and total intensity of mature pre- and post-synaptic spot size without affecting the mean intensity. These results imply that under the current conditions there is a narrow window for effects of FC or solvents supplementation on synapse morphology *in vitro*.

**Figure 9:**
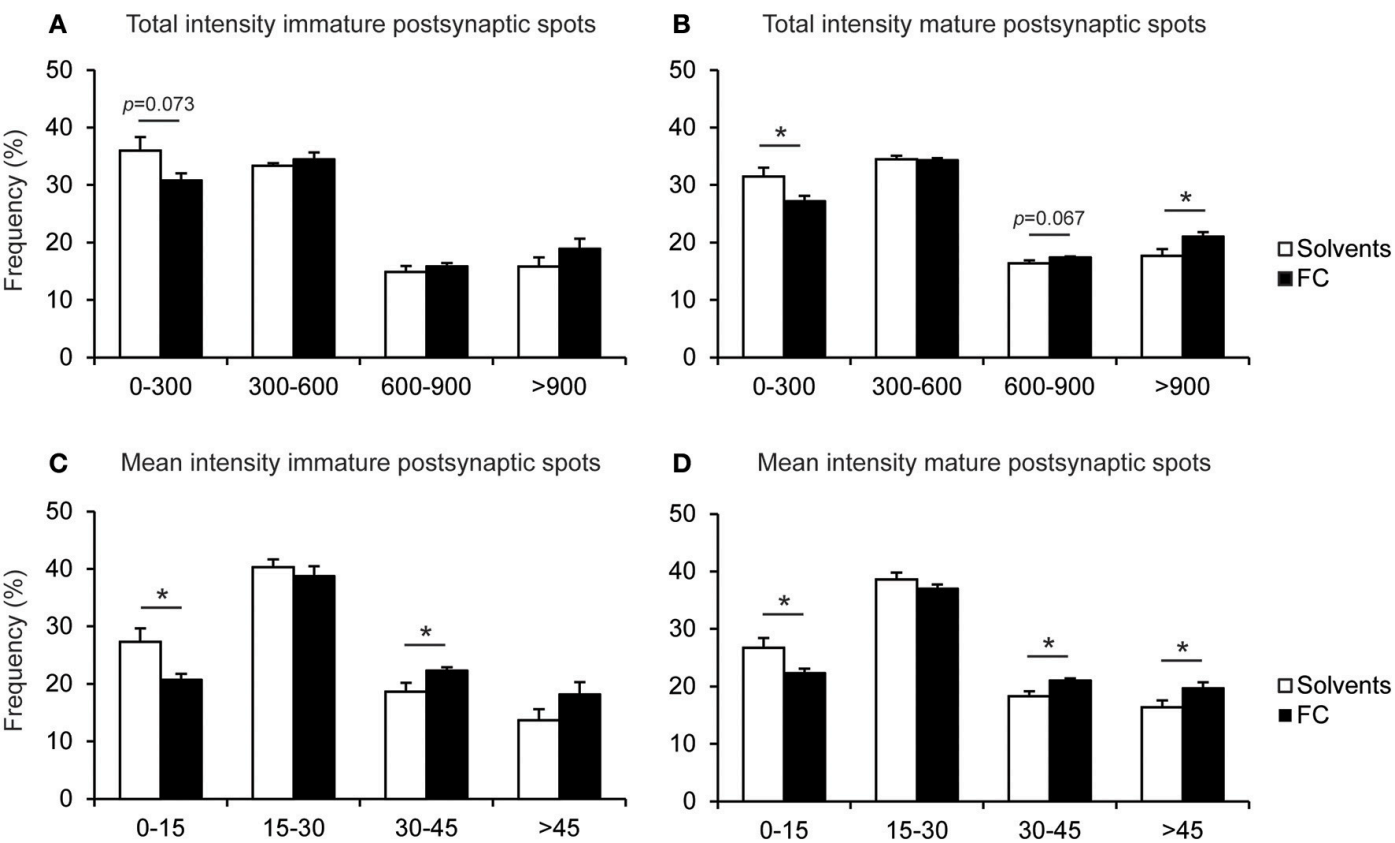
Effect of 0.05x FC supplementation on the PSD95-intensity of immature and mature postsynaptic spots. Frequency distribution of the total PSD95 intensity of individual immature postsynaptic spots **(A)** and mature postsynaptic spots **(B)**. Frequency distribution of the mean intensity (intensity corrected for spot size) of individual immature postsynaptic spots **(C)** and mature postsynaptic spots **(D)**. Error bars represent standard error of the mean (SEM), *n* = 8 wells per condition, ^*^p < 0.05.

Since the intensity of synaptic protein stainings may be related to maturation or strength of the synapse (Petralia et al., 2005), and the total population of synapses is likely to include synapses with different maturation levels (Bartol et al., 2015), we evaluated whether 0.05x FC supplementation caused a shift in possible synapse populations with certain postsynaptic spot intensity levels. We found that 0.05x FC caused a shift toward increased total and mean intensity of both mature and immature postsynaptic spots (Figure 9), which is in line with the increased intensity of postsynaptic spots after 0.05x FC supplementation, as shown in Figure 8.

## Discussion

In the current study, we used a new method of analysis to examine the effect of nutritional phospholipid precursors and cofactors, as provided by Fortasyn Connect (FC) supplementation, on synapse formation and maturation of primary hippocampal neurons co-cultured with astrocytes. We found that FC increased neuronal survival and the maturation of postsynaptic terminals.

### New method of analysis for neuronal developmental processes in neuron-astrocyte co-cultures

Using the Opera High Content Screening system and Columbus analysis, we were able to measure neuronal survival, neurite morphology, synapse formation and maturation in neuron-astrocyte co-cultures in an automated manner. Using a low magnification (20x), accurate categorization of neuronal and astrocyte nuclei, tracing of even thin and less intense MAP2-positive neurites and synapse count was performed in an automated fashion. In this way, synapsin1-positive presynaptic terminals and PSD95-positive postsynaptic terminals were detected along the entire neurite tree. Imaging at a higher magnification (40x) enabled us to analyze synapse size and intensity, again in an automated fashion. It should be noted that we mainly focused on proximal synapses since synapse detection was performed along neurites protruding from the soma whereas the entire neurite tree was often not captured within one single 40x image. We found that under basal culture conditions, the majority (71%) of synapses were mature, i.e., consisting of opposing pre- and post-synaptic terminals. Also immature synaptic terminals were detected, consisting of either non-opposing presynaptic (20%) or postsynaptic (9%) terminals along neurites. It should also be noted that images were taken at a fixed focal plane, which could have increased the variability in measured terminal size and intensity and resulted in overestimation of the number of immature terminals, because possible opposed terminals were present in different focal planes and therefore remained undetected.

Interestingly, we were able to detect differences between the morphology of immature versus mature synapses. Mature presynaptic terminals were larger and contained higher synapsin1 levels than immature presynaptic terminals. Likewise, mature postsynaptic terminals contained higher PSD95 levels compared to immature postsynaptic terminals. Taken together, this suggests that mature synapses consist of enlarged presynaptic terminals with more synapsin1 and of postsynaptic terminals with more scaffolding protein PSD95 than immature terminals. This is in line with previous *in vitro and in vivo* studies showing that synaptic maturation is accompanied by an increase in presynaptic terminal size and vesicle number, as well as increased levels of PSD95 (Dyson and Jones, 1980; Fletcher et al., 1991; Ziv and Garner, 2004; Petralia et al., 2005). Furthermore, PSD95 can enhance the synaptic localization of glutamate receptors leading to increased synaptic activity (El-Husseini et al., 2000). The ability to measure the development of pre- and post-synaptic terminals into mature synapses in neuron-astrocyte co-cultures in an automated and high throughput fashion enables assaying intra- and extra-cellular factors, such as nutritional compounds, affecting these processes. Our HCS of synapse formation and maturation using immunostainings may, in the future, be complemented with the use of fluorescent dyes to visualize synapse activity. Currently available dyes are suitable for analysis of synaptic endocytosis and exocytosis; however, require live imaging next to induced activation of synaptic activity, which so far is not compatible with current HCS devices.

### Effect of FC on neuronal developmental processes

We investigated the effect of FC on neuronal developmental processes of primary hippocampal neurons co-cultured with primary astrocytes by supplementing FC at a developmental time point *in vitro* when most neurites had been formed and synaptogenesis occurred (Pennypacker et al., 1991; Kim and Lee, 2012; Harrill et al., 2015), thereby focusing on the effect of FC on synapse formation and maturation. Since FC components were dissolved in fatty acid free bovine serum albumin (FAF-BSA) and ethanol, these solvents at different dilutions corresponding to FC dilutions were studied in parallel as appropriate control. Supplementation with solvents already enhanced mature pre- and post-synaptic terminal size accompanied by increased levels of synapsin1 and PSD95, most likely because of the presence of FAF-BSA. Previous studies showed that albumin promotes neuronal differentiation by stimulating fatty acid synthesis by astrocytes (Tabernero et al., 2001; Medina and Tabernero, 2002). Therefore, the results of supplementation with FC were primarily compared to their corresponding solvent condition. Given that solvents had positive effects on synaptic terminal morphology in the current *in vitro* assay, the effect that FC has on synapse formation and maturation *in vivo* is likely to be underestimated by the present findings.

Using our current experimental conditions, FC increased neuronal survival in a dose-dependent manner, without affecting neurite morphology. Indeed, no effects on neurite outgrowth were expected in the current set up since FC was supplemented after most of the neurite outgrowth had taken place. The finding that FC increased neuronal survival suggests that FC has a neuroprotective effect, which is in line with previous *in vivo* findings (Zerbi et al., 2014). It should be noted that the neuroprotective effect in the current experiment led to a strong increase in the number of neurons per well, and therefore in the number of neurites and synapses per well, which may have decreased the effects of FC supplementation on neurite and synapse number. Despite this, a small positive effect of FC on the number of immature presynaptic terminals was found.

Interestingly, it was found that FC had a positive effect on the maturation of synapses. Low concentrations of FC significantly increased the PSD95 signal intensity in mature terminals and possibly also immature postsynaptic terminals, suggesting that FC actively increased the level of PSD95 protein in postsynaptic terminals. Given that mature postsynaptic terminals contained more PSD95 protein than immature postsynaptic terminals under basal culture conditions, this implied that FC induced postsynaptic maturation. No effects of FC on presynaptic synapsin1 levels were observed, suggesting that the maturation of the presynaptic terminal is not affected. Taken together, we showed that FC has positive effects on the maturation of postsynapses in our co-culture when supplemented within a specific range.

Given that synapses with increased PSD95 levels contain more glutamate receptors and have enhanced glutamate receptor activity (Takumi et al., 1999; El-Husseini et al., 2000), these findings suggest that FC could increase hippocampal neurotransmission and plasticity, which may eventually result in a further increased number of synapses *in vivo*, since stronger and more active synapses survive for a longer period of time (Stephan et al., 2012; Hong et al., 2016). This adds to previous findings that chronic supplementation with DHA and UMP to healthy rats and gerbils had positive effects on hippocampal spine density (Sakamoto et al., 2007; Cansev et al., 2009).

### Implications for the roles of FC and astrocytes in neurodevelopmental disorders

Including astrocytes in cell culture experiments is important because of their metabolic role in neuronal functioning (Camargo et al., 2009; Bélanger et al., 2011). Astrocytes regulate processes, such as neuronal survival, synapse formation and synaptic transmission by secreting gliotransmitters including glutamate, adenosine, D-serine and ATP, but also lactate and lipids (reviewed in Araque et al., 1999; Allen, 2014). In the present study, no effect of FC on astrocyte number was observed. Since astrocytes have positive effects on synapse formation and function, the effect of FC might be complementary. In addition, FC may affect astrocyte activity, without affecting astrocyte number, which might (partially) underlie the observed increase in neuronal and synaptic support. Indeed, the FC component DHA was demonstrated to affect glial activity under conditions of neuroinflammation *in vivo* (Lu et al., 2013). Studying the effect of FC on developmental processes of neurons co-cultured under disease conditions would provide further insight into the role of astrocytes as well as the effect of FC under pathological conditions. This high content screening method could be used to assess the effect of various pharmacological and nutritional treatments, on synaptogenesis of disease state neurons. This may be of great benefit to the design of intervention strategies of patients suffering from impaired neuronal development or synaptic loss, such as in AD. Previous studies reported that FC, present in the medical food Souvenaid, improved memory performance in patients with early AD, possibly by influencing functional connectivity (Scheltens et al., 2010, 2012; De Waal et al., 2014). Here, it has been shown that FC has a positive effect on synapse maturation. Making synapses stronger might be a mechanism to prevent their dysfunction and loss (Hong et al., 2016).

Taken together, the present results show that FC supplementation to primary neuron-astrocyte co-cultures enhances neuronal survival and increases the expression of postsynaptic protein PSD95 in mature and immature synapses. These findings might have important implications for the understanding of intervention strategies in neurological diseases characterized by impaired synaptic functioning.

## Author contributions

AvD, LB, JV, and MV designed research; AvD and MV performed research; LB and JV contributed new reagents/analytic tools; AvD and MV analyzed data; and AvD, LB, JV, MV, and AS wrote the paper.

### Conflict of interest statement

LB and JV are employees of Nutricia Research. The other authors declare that the research was conducted in the absence of any commercial or financial relationships that could be construed as a potential conflict of interest.
